# The Ambulance Attendant: A Curious Case

**Published:** 1914-07-25

**Authors:** 


### The Ambulance Attendant: A Curious Case.

A well-known decision on the Workmen's Compensation Act, and one of considerable medical interest, was "the aneurism case" (Clover, Clayton & Co. [1910] A.C. 242). There a man who was suffering from a serious aneurism suddenly fell down dead from rupture of the aneurism while he was tightening a nut by means of a spanner. The man at the time was engaged in his ordinary work, and according to the evidence might have died in his sleep from the same cause, although the strain which he experienced while screwing the nut probably accelerated the rupture. The House of Lords held that the workman had met his death by accident. That decision has just been followed in a case which, both as regards the facts and in its medical aspects, is of interest. A workman was employed as a gate-man at a factory. His duties were to attend to the gate and take charge of the ambulance appliances, etc., for use in case of accidents occurring in the works, to telephone for the doctor or ambulance should necessity arise, to attend personally to any minor accidents, and generally to attend to the telephone in the morning before any of the office staff arrived. One morning about 7.30 a.m., and before the office staff arrived, he was informed of a scaffold accident which had happened to some slaters who were making some repairs on the factory premises. On being so informed he ran from the gate to the scene of the accident, a distance of about a hundred yards, and ran back to the gate in order to telephone for a doctor and an ambulance; and while engaged telephoning he was seized with an apoplectic shock which terminated fatally about 1.30 p.m. on the same day. These circumstances raised two questions: (1) Did the deceased meet his death by accident? And (2) even though the occurrence might be termed an accident, did it arise out of and in course of the man's employment? An affirmative answer to both these questions was necessary in order to entitle his dependants to the benefit of compensation. The Court of Appeal, reversing the judgment of the Court below, have found in the affirmative on both points, on the ground that the apoplectic seizure was the direct effect of an abnormal effort made while on duty.

